# Homocysteine Levels in Patients with Schizophrenia on Clozapine Monotherapy

**DOI:** 10.1007/s11064-013-1113-1

**Published:** 2013-07-20

**Authors:** Adam Wysokiński, Iwona Kłoszewska

**Affiliations:** Department of Old Age Psychiatry and Psychotic Disorders, Medical University of Lodz, Czechosłowacka 8/10, 92-216 Łódź, Poland

**Keywords:** Schizophrenia, Homocysteine, Clozapine

## Abstract

We tested the hypothesis that homocysteine levels are higher in blood of schizophrenic subjects on clozapine monotherapy than in healthy controls and they correlate with anthropometric measurements, laboratory tests and results of bioimpedance analysis of body composition. Data for 24 subjects with schizophrenia treated with clozapine and 24 age- and sex-matched healthy volunteers was analyzed. Regarding the whole group, homocysteine levels were significantly higher in men (17.0 ± 3.4 vs. 12.1 ± 4.0 μmol/L, *p* = 0.009). Homocysteine levels correlated with waist circumference (R = 0.58, *p* = 0.003), waist-to-hip ratio (R = 0.57, *p* = 0.003), basal metabolic rate (R = 0.48, *p* = 0.01), lean body mass [kg] (R = 0.53, *p* = 0.008), body water [L] (R = 0.53, *p* = 0.008) and triglycerides (R = 0.57, *p* = 0.003). There were no significant differences of homocysteine levels for impaired fasting glucose, abdominal obesity, obesity/overweight, and dyslipidemia. Homocysteine levels did not correlate with age, treatment duration, clozapine dose, weight, body mass index, abdominal circumference, blood pressure, total body fat, cholesterol, high density lipoproteins, low density lipoproteins, uric acid, calcium, glucose, insulin, homoeostasis model assessment of insulin resistance 1, and homoeostasis model assessment of insulin resistance 2. We did not find significant differences in blood homocysteine levels between subjects with schizophrenia and controls. Association with waist circumference may support homocysteine role as an important cardiovascular risk factor. Association with lean weight may explain why men have higher levels of homocysteine than women.

## Introduction

Homocysteine is an amino-acid produced during demethylation of methionine. High levels of homocysteine in blood are associated with increased risk of cardiovascular disease (CVD) [1]. Patients with schizophrenia may have increased levels of homocysteine [2] and this may add to increased CVD risk due to treatment with antipsychotics [3]. Homocysteine concentration is inversely related to the intake and plasma levels of folate [4], while folate deficiency is common in schizophrenic patients [5]. As it was shown by Lee et al. [6], raised homocysteine levels may also result from the 677TT genotype of methylenetetrahydrofolate reductase (MTHFR), enzyme that catalyzes the conversion of 5,10-methylenetetrahydrofolate to 5-methyltetrahydrofolate, a cosubstrate for homocysteine remethylation to methionine. Homocysteine may trigger neuronal apoptosis [7], promote oxidative stress [8] and induce neurotoxicity via the N-methyl-D-aspartate (NMDA) receptor [9]. It may also increase the risk of tardive dyskinesia in schizophrenia patients [10]. Levine et al. [11] showed that simple interventions aimed at reducing homocysteine levels (supplementation with B vitamins) may improve clinical and cognitive symptoms in chronic schizophrenic patients with hyperhomocysteinemia.

After more than 50 years since it has been discovered, clozapine remains an ultimate option for patients with treatment resistant schizophrenia [12]. Its high efficacy is combined with very low level of extrapyramidal symptoms and ability to ameliorate tardive dyskinesia. However, treatment with clozapine is associated with increased risk of fatal agranulocytosis and has also a very detrimental effect on metabolic profile [13], which may contribute to dramatically increased mortality of schizophrenia patients [14]. Clozapine has a very distinctive pharmacological profile, which probably underlies its efficacy and side-effects [15]. Apart from its affinity to various receptors for monoamine neurotransmitters, its has several unique properties, some of them are linked to actions induced by homocysteine. Clozapine blocks neurotoxicity in the rat cortex induced by noncompetitive NMDA receptor antagonist dizocilpine [16]. It may also provide some degree of neuroprotection, specifically against oxidative stress [17]. Using an animal model, Moller et al. [18] showed that clozapine may reverse cortico-striatal oxidative stress. However, it was also demonstrated by Fehsel et al. [19] that clozapine induces oxidative stress in neutrophils, which may trigger agranulocystosis.

## Aims of the Study

The present study was undertaken with the purpose to determine whether subjects on monotherapy with clozapine have higher levels of blood homocysteine comparing to healthy control. In order to provide more accurate measurements, biochemical and anthropometric measurements were combined with body composition determined using bioelectric impedance analysis (BIA), which provides accurate measurements of body fat, lean mass and body water [20]. To the best of our knowledge, this is the first study to investigate such combination of these parameters in subjects with schizophrenia.

## Materials and Methods

Data for 24 European Caucasian adult patients with paranoid schizophrenia (295.30, according to DSM-IV) was included into the study. These subjects were on clozapine monotherapy for at least 2 months prior the assessments. Control group was 24 healthy subjects and was gender- and age-matched with patients in the clozapine group. All patients and volunteers included in the study have been informed about aims and methods of the study and expressed their written informed consent for participation in this study. The study protocol was approved by the local Bioethics Committee. There was no financial involvement from the industry.

### Laboratory Tests

The blood samples for the chemistry panel were collected between 7 am and 8 am, after ensuring at least 8 h of overnight fasting. The samples were immediately transferred to the central laboratory where they were analyzed. Glucose, lipids, calcium and uric acid levels were measured using a Dirui CS-400 analyzer (Dirui, China). Homocysteine chemiluminescence assessments were performed using an Immulite 2000 analyzer (Siemens, Germany), insulin immunochemistry assessments were performed using a Cobas E411 analyzer (Roche Diagnostics, Switzerland) and albumin levels were assessed using a Cobas Integra 800 analyzer (Roche Diagnostics, Switzerland).

Impaired fasting glucose was defined as fasting plasma glucose ≥100 mg/dL. BMI <25 kg/m^2^, 25–30 kg/m^2^ and ≥30 kg/m^2^ were defined as normal weight, overweight and obesity, respectively. Raised triglycerides (TGA) level ≥150 mg/dL and/or total cholesterol (TC) ≥200 mg/dL and/or reduced HDL cholesterol level <40 mg/dL for men and <50 mg/dL for women and/or raised LDL cholesterol level ≥135 mg/dL were interpreted as dyslipidemia. Corrected calcium was calculated using the formula: corrected calcium (mg/dL) = measured total calcium (mg/dL) + 0.8 (4.0 − serum albumin [g/dL]). Insulin resistance was estimated from fasting glucose and insulin results by homeostasis model assessment, using the formula: HOMA1-IR = (fasting plasma glucose [mg/dL] × insulin [mU/L])/405. HOMA2-IR index was calculated using a calculator downloaded from http://www.dtu.ox.ac.uk.

### Anthropometric Assessment

Height was measured with a wall-mounted height measure to the nearest 0.5 cm. Weight was measured with a spring balance that was kept on a firm horizontal surface. Subjects wore light clothing, stood upright without shoes and weight was recorded to the nearest 0.5 kg. Body mass index (BMI) was calculated as body weight in kilogram divided by the height in meter squared (kg/m^2^). Waist, abdominal and hip circumference was measured using a non-stretchable fibre measuring tape.

### Body Composition Assessment

Body composition was measured using a Maltron BF-906 body fat analyser (Maltron, UK), single frequency bioelectrical impedance analyser to determine resistance and reactance at 50 Hz. Standard operating conditions were observed by a trained operator including preparation of the participant, electrode placement and operation. The measurement using BIA was taken immediately prior to anthropometry measurements with participants lying supine, in a rested state.

### Statistical Methods

Statistical procedures were performed with STATA 12.1 for OS X (StataCorp, College Station, Texas, USA). Simple descriptive statistics (means and standard deviations, median (Q2), 25 and 75 % quartiles (Q1 and Q3)) were generated for all continuous variables. For discrete variables number of patients and percentages are given. Inter-group differences were analyzed using Mann–Whitney *U* test. The difference between proportions was analyzed by Fisher’s exact test. Associations were tested by Spearman’s correlation coefficient. The significant level was set at *p* < 0.05.

## Results

For group of patients treated with clozapine the mean age was 38.8 ± 12.6 [Q1 = 28.0, Q2 = 38.5, Q3 = 47.5] and 39.9 ± 12.3 [Q1 = 30.5, Q2 = 36.0, Q3 = 52.0] for the control group; there was no significant difference between the groups in age (*p* = 0.62). In both groups there were 12 men, i.e. half of group, and 12 women. In the clozapine group 12 (half of group) subjects smoked cigarettes and 8 in the control group (*p* = 0.38). The mean duration of monotherapy with clozapine was 131.8 ± 114.3 [Q1 = 8.5, Q2 = 33.0, Q3 = 84.0] months and mean clozapine dose was 341.1 ± 148.6 [Q1 = 237.5, Q2 = 300.0, Q3 = 425.0] mg/day. Detailed results for anthropometric measurements and laboratory tests are shown in Table 1. We have found no inter-group differences for body composition analysis. Detailed results for BIA analysis are shown in Table 2. Lean body mass was higher in men in the whole study sample (60.1 ± 6.4 [Q1 = 53.8, Q2 = 59.7, Q3 = 63.6] vs. 43.8 ± 5.4 kg [Q1 = 41.3, Q2 = 43.9, Q3 = 46.4], z = −5.74, *p* < 0.001) and in the clozapine group (59.6 ± 5.7 [Q1 = 55.3, Q2 = 59.7, Q3 = 61.4] vs. 45.3 ± 7.0 kg [Q1 = 42.5, Q2 = 46.4, Q3 = 49.1], z = −3.93, *p* < 0.001). Similarly, basal metabolic rate was higher in men in the whole study sample (1,707.7 ± 182.3 [Q1 = 1,567.0, Q2 = 1,731.0, Q3 = 1,837.0] vs. 1,337.3 ± 138.4 [Q1 = 1,229.5, Q2 = 1,380.5, Q3 = 1,389.0] kg, z = −5.32, *p* < 0.001) and in the clozapine group (1,701.2 ± 138.2 [Q1 = 1,582.0, Q2 = 1,722.5, Q3 = 1,790.0] vs. 1,362.7 ± 173.0 [Q1 = 1,281.5, Q2 = 1,388.5, Q3 = 1,465.0] kg, z = −3.87, *p* < 0.001).

**Table 1:** Results of anthropometric measurements and laboratory tests

	Clozapine (n = 24)	Control (n = 24)	*p*
BMI (kg/m^2^)	27.1 ± 3.6 (25.1, 26.2, 30.7)	24.8 ± 3.5 (22.8, 24.8, 26.8)	z = −2.16 *p* = 0.03
Abdominal circumference (cm)	96.5 ± 9.4 (89.0, 97.0, 103.5)	85.5 ± 11.6 (79.5, 86.0, 93.0)	z = −3.37 *p* < 0.001
Waist circumference (cm)	91.1 ± 12.1 (82.5, 90.5, 100.5)	82.4 ± 10.6 (75.0, 83.0, 89.5)	z = −2.52 *p* = 0.01
WHR	0.92 ± 0.08 (0.87, 0.92, 0.98)	0.86 ± 0.08 (0.79, 0.86, 0.90)	z = 2.55 *p* = 0.01
SBP (mm Hg)	121.7 ± 13.6 (114.5, 123.5, 129.5)	136.7 ± 17.9 (123.5, 138.5, 151.5)	z = 2.98 *p* = 0.003
DBP (mm Hg)	81.2 ± 8.5 (75.5, 80.0, 86.0)	82.8 ± 12.1 (73.5, 83.5, 89.5)	*p* = 0.60
Homocysteine (μmol/L)	14.5 ± 4.4 (11.3, 15.5, 17.7)	13.6 ± 5.0 (10.2, 13.5, 16.5)	*p* = 0.48
TC (mg/dL)	194.2 ± 53.2 (157.2, 184.4, 214.6)	216.6 ± 65.3 (174.3, 205.8, 238.1)	*p* = 0.17
HDL (mg/dL)	43.5 ± 12.6 (35.0, 39.9, 53.3)	55.1 ± 14.3 (46.3, 57.1, 64.6)	z = 2.69 *p* = 0.007
LDL (mg/dL)	122.6 ± 41.9 (90.9, 115.4, 154.8)	128.3 ± 39.7 (94.6, 123.4, 167.3)	*p* = 0.59
TGA (mg/dL)	140.3 ± 120.4 (74.8, 105.1, 182.6)	104.3 ± 81.4 (57.1, 85.4, 127.6)	*p* = 0.19
FPG (mg/dL)	103.5 ± 31.7 (87.4, 94.8, 112.9)	87.8 ± 11.7 (81.2, 85.9, 95.9)	z = −2.03 *p* = 0.04
Insulin (μg/mL)	11.8 ± 8.2 (6.7, 8.7, 12.3)	7.7 ± 3.2 (5.6, 7.0, 9.0)	*p* = 0.08
HOMA1-IR	3.3 ± 3.4 (1.5, 2.0, 3.7)	1.7 ± 0.8 (1.2, 1.6, 2.1)	*p* = 0.06
HOMA2-IR	1.6 ± 1.1 (0.9, 1.0, 1.7)	1.0 ± 0.4 (0.7, 0.9, 1.2)	*p* = 0.06
Albumin (g/dL)	4.5 ± 0.5 (4.3, 4.5, 4.9)	4.7 ± 0.3 (4.5, 4.6, 4.8)	*p* = 0.20
Total calcium (mg/dL)	9.0 ± 0.8 (8.5, 9.0, 9.6)	9.3 ± 0.7 (8.7, 9.3, 9.7)	*p* = 0.39
Corrected calcium (mg/dL)	8.6 ± 0.9 (8.0, 8.7, 9.4)	8.7 ± 0.7 (8.3, 8.7, 9.3)	*p* = 0.94
Uric acid (mg/dL)	4.5 ± 1.4 (3.5, 4.2, 5.0)	4.3 ± 1.3 (3.8, 4.4, 4.9)	*p* = 0.86

Data given as mean ± standard deviation (25 % quartile, median, 75 % quartile)

*BMI* body mass index, *WHR* waist-to-hip ratio, *SBP* systolic blood pressure, *DBP* diastolic blood pressure, *TC* total cholesterol, *HDL* high density lipoproteins, *LDL* low density lipoproteins, *TGA* triglycerides, *FPG* fasting plasma glucose, *HOMA1-IR* homoeostasis model assessment of insulin resistance 1, *HOMA2-IR* homoeostasis model assessment of insulin resistance 2

**Table 2:** Results of body composition analysis

	Clozapine (n = 24)	Control (n = 24)	*P*
Total body fat (%)	32.6 ± 8.4 (29.0, 33.2, 38.5)	28.9 ± 7.1 (24.2, 28.9, 33.0)	*p* = 0.06
Total body fat (kg)	25.6 ± 8.8 (20.1, 24.5, 31.5)	22.4 ± 8.7 (18.3, 21.2, 26.2)	*p* = 0.12
Target body fat min (%)	21.6 ± 4.0 (20.0, 20.0, 24.0)	22.2 ± 3.6 (19.0, 22.5, 25.0)	*p* = 0.57
Target body fat max (%)	27.6 ± 4.0 (26.0, 26.0, 30.0)	28.2 ± 3.6 (25.0, 28.5, 31.0)	*p* = 0.57
Basal metabolic rate (kcal/day)	1,532.0 ± 230.9 (1,388.5, 1,527.0, 1,722.5)	1,513.0 ± 265.4 (1,347.0, 1,386.5, 1,731.5)	*p* = 0.39
Target weight min (kg)	58.0 ± 9.1 (53.5, 58.0, 64.0)	56.6 ± 10.1 (49.0, 53.0, 66.0)	*p* = 0.29
Target weight max (kg)	69.0 ± 10.3 (64.5, 69.0, 74.0)	67.9 ± 11.2 (59.5, 63.5, 78.0)	*p* = 0.41
Lean body weight (kg)	52.5 ± 9.6 (61.5, 66.8, 70.9)	51.4 ± 10.8 (67.0, 71.0, 75.8)	*p* = 0.48
Lean body weight (%)	67.6 ± 8.1 (46.4, 53.0, 59.7)	71.1 ± 7.1 (42.5, 47.7, 61.0)	*p* = 0.07
Total body water (L)	38.4 ± 7.0 (33.9, 38.8, 43.7)	37.7 ± 7.9 (31.1, 34.9, 44.6)	*p* = 0.48
Total body water (%)	49.9 ± 5.4 (46.5, 48.9, 52.0)	52.1 ± 5.2 (49.0, 52.0, 55.5)	*p* = 0.09
Target body water min (%)	50.7 ± 3.2 (49.0, 52.0, 52.0)	50.1 ± 2.9 (48.0, 50.0, 53.0)	*p* = 0.57
Target body water max (%)	57.7 ± 3.2 (56.0, 59.0, 59.0)	57.3 ± 2.8 (55.0, 57.0, 60.0)	*p* = 0.66

Data given as mean ± standard deviation (25 % quartile, median, 75 % quartile)

In the clozapine group fasting homocysteine levels correlated with waist circumference (R = 0.58, *p* = 0.003), waist-to-hip ratio (R = 0.57, *p* = 0.003), basal metabolic rate (R = 0.48, *p* = 0.01), lean body mass [kg] (R = 0.53, *p* = 0.008), body water [L] (R = 0.53, *p* = 0.008) and TGA (R = 0.57, *p* = 0.003). Fasting serum homocysteine concentration did not correlate with age (R = 0.24, *p* = 0.26), duration of clozapine treatment (R < 0.01, *p* = 0.98), clozapine dose (R = 0.02, *p* = 0.91), weight (R = 0.38, *p* = 0.07), BMI (R = 0.25, *p* = 0.23), abdominal circumference (R = 0.35, *p* = 0.09), systolic blood pressure (R = 0.06, *p* = 0.77), diastolic blood pressure (R = 0.09, *p* = 0.66), total body fat [kg] (R = 0.13, *p* = 0.56), TC (R = 0.38, *p* = 0.7), HDL (R = −0.38, *p* = 0.06), LDL (R = 0.39, *p* = 0.06), uric acid (R = 0.29, *p* = 0.17), corrected calcium (R = −0.12, *p* = 0.57), glucose (R = −0.07, *p* = 0.76), insulin (R = 0.04, *p* = 0.83), HOMA1-IR (R = −0.16, *p* = 0.45), HOMA2-IR (R = −0.01, *p* = 0.97).

The normal clinical laboratory range for homocysteine was 5.0–12.0 μmol/L. There was no difference of fasting homocysteine concentrations between clozapine and control group (14.5 ± 4.4 [Q1 = 11.3, Q2 = 15.5, Q3 = 17.7] vs. 13.6 ± 5.0 [Q1 = 10.2, Q2 = 13.5, Q3 = 16.5] μmol/L, *p* = 0.48). In the clozapine group homocysteine levels were significantly higher in men than in women (17.0 ± 3.4 [Q1 = 15.4, Q2 = 17.2, Q3 = 18.5] vs. 12.1 ± 4.0 [Q1 = 8.1, Q2 = 11.9, Q3 = 15.5] μmol/L, z = −2.63, *p* = 0.009). The difference between men and women was also significant for the whole study population (16.5 ± 3.4 [Q1 = 14.0, Q2 = 17.0, Q3 = 18.2] vs. 11.6 ± 4.6 [Q1 = 8.1, Q2 = 11.8, Q3 = 14.4] μmol/L, z = −3.69, *p* < 0.001). Blood homocysteine levels were not significantly different between men with schizophrenia and healthy men (17.0 ± 3.4 [Q1 = 15.4, Q2 = 17.2, Q3 = 18.5] vs. 16.0 ± 3.5 [Q1 = 11.8, Q2 = 13.7, Q3 = 14.4] μmol/L, *p* = 0.4) and between women with schizophrenia and healthy women (12.1 ± 4.0 [Q1 = 8.1, Q2 = 11.9, Q3 = 15.5] vs. 11.2 ± 5.2 [Q1 = 8.0, Q2 = 10.6, Q3 = 13.2] μmol/L, *p* = 0.6). In the clozapine group there were no significant differences between smokers and non-smokers (13.6 ± 4.8 [Q1 = 9.0, Q2 = 14.2, Q3 = 16.7] vs. 15.5 ± 3.9 [Q1 = 11.9, Q2 = 16.2, Q3 = 18.5] μmol/L, *p* = 0.21), subjects with normal and impaired fasting glucose (14.9 ± 4.6 [Q1 = 11.5, Q2 = 15.3, Q3 = 16.2] vs. 14.1 ± 4.53 [Q1 = 8.2, Q2 = 16.8, Q3 = 18.2] μmol/L, *p* = 0.98), subjects with total body fat lower and higher than target maximum based on BIA (14.4 ± 4.3 [Q1 = 10.7, Q2 = 15.7, Q3 = 17.7] vs. 15.2 ± 5.4 [Q1 = 11.4, Q2 = 13.4, Q3 = 19.0] μmol/L, *p* = 0.93), subjects with BMI <25 and ≥25 kg/m^2^ (13.6 ± 5.2 [Q1 = 11.5, Q2 = 12.4, Q3 = 15.2] vs. 14.8 ± 4.2 [Q1 = 11.2, Q2 = 15.9, Q3 = 18.2] μmol/L, *p* = 0.93), without abdominal obesity and with abdominal obesity (15.4 ± 4.2 [Q1 = 11.6, Q2 = 15.7, Q3 = 18.2] vs. 12.4 ± 4.3 [Q1 = 8.2, Q2 = 12.2, Q3 = 16.2] μmol/L, *p* = 0.19), without and with dyslipidemia (12.6 ± 5.0 [Q1 = 8.2, Q2 = 11.5, Q3 = 15.3] vs. 15.7 ± 3.7 [Q1 = 13.3, Q2 = 16.2, Q3 = 18.3] μmol/L, *p* = 0.08). No such differences were also found for the whole study group.

## Discussion

Contrary to other observations [2], we did not find that subjects with schizophrenia on clozapine monotherapy had higher fasting homocysteine levels comparing to age- and sex-matched healthy controls. However, using two-sample mean-comparison calculator we have found that homocysteine levels in men were not significantly different than reported by Levine et al. (16.3 ± 11.8 μmol/L; *p* = 0.42) [21] and significantly higher then reported by Henderson et al. (7.69 ± 1.42 μmol/L; t_51_ = −14.06, *p* < 0.001) [22]. It should be noted that subjects in the study of Henderson et al. had schizophrenia or schizoaffective disorder and were taking clozapine, risperidone or olanzapine as monotherapy. No details for antipsychotic treatment were given by Levine et al. in their paper. Therefore, we may assume that homocysteine levels in our patients were relatively high, particularly in men. Considering atherogenic properties of homocysteine, this is important since in many cases treatment with clozapine is associated with metabolic side-effects significantly increasing the risk of cardiovascular events. We have confirmed previous observations that fasting homocysteine levels are higher in men than in women, both in the clozapine group, as well as in the whole study population. It was previously reported by Sanchez-Margalet et al. [23] that obese subjects have higher levels of homocysteine. We have found no such associations for abdominal obesity, general obesity (determined using BMI-based cut-off values) and in subjects with total body fat higher than target maximum calculated using BIA.

Homocysteine levels were positively correlated with waist circumference and WHR, but not with BMI. Considering the association between hyperhomocysteinemia and raised risk of CVD, this observation is consistent with previous reports that WHR is the best predictor of CVD risk, premature death, stroke, non-insulin-dependent diabetes mellitus and female carcinomas [24], while BMI is negatively correlated to cardiovascular disease, premature death, and stroke, but positively to diabetes [25]. Lin et al. [26] has shown that in male patients with coronary artery disease homocysteine levels are strongly associated with WHR, but not with BMI. Association between homocysteine and TGA blood levels also indicates a potential impact of homocysteine on CVD risk.

In our study fasting homocysteine levels were positively correlated with lean body mass and body water. Similar results were previously reported for healthy subjects by Battezzati et al. [27]. Several mechanisms explaining this association were proposed (e.g. amount of fat-free mass determines creatine synthesis, which is the single quantitatively most important biological reaction requiring methyl groups from methionine to produce homocysteine or the fact that the level of creatinine, whose production is related to fat-free mass, is positively related to the homocysteine concentration). We have found that both in the clozapine group and in the whole study population men had higher amount of lean body mass, which may explain why homocysteine levels are higher in men. The same reasoning could be used to explain observed association between homocysteine levels and basal metabolic rate, since it was also significantly higher in men than in women.

Finally, apart from TGA, we have found no other associations of homocysteine blood levels with any biochemical variable analyzed. Contrary to Henderson et al. [22], we also have found no differences between subjects with normal and impaired fasting blood glucose. However, we were not able to stratify our study sample by folate intake or levels, which was done by these authors. Since they found that folate effects may differ depending on fasting glucose status, we cannon exclude that different folate levels in our subjects could have resulted in the absence of such association. Our observations are similar to previously reported by Abbasi et al. [28], who found no relationship between insulin resistance and plasma homocysteine concentrations in a group of healthy volunteers.

## Conclusions

There was no significant difference in blood homocysteine levels between patients wit schizophrenia treated with clozapine and mentally healthy controls. Men with schizophrenia taking clozapine had a significantly higher homocysteine levels than women. Homocysteine levels were positively correlated with waist circumference, WHR, TGA levels, basal metabolic rate, lean weight and body water.

Low number of study subjects limited the probability of finding inter-group differences due to lack of statistical power. Due to the cross-sectional study design causal relationships cannot be established and effect of previous antipsychotic treatment cannot be excluded. Dual-energy X-ray absorptiometry (DXA) could be used to measure body composition and percentage of fat more accurately.

## Abbreviations

CVD: Cardio-vascular disease
BIA: Body impedance analysis
BMI: Body mass index
WHR: Waist-to-hip ratio
SBP: Systolic blood pressure
DBP: Diastolic blood pressure
TC: Total cholesterol
HDL: High density lipoproteins
LDL: Low density lipoproteins
TGA: Triglycerides
FPG: Fasting plasma glucose
HOMA1-IR: Homoeostasis model assessment of insulin resistance 1
HOMA2-IR: Homoeostasis model assessment of insulin resistance 2

## References

[1] Marti-Carvajal AJ, Sola I, Lathyris D, Salanti G (2009) Homocysteine lowering interventions for preventing cardiovascular events. Cochrane Database Syst Rev CD006612 10.1002/14651858.CD006612.pub2 PMC4164174 19821378

[2] Neeman G, Blanaru M, Bloch B, Kremer I, Ermilov M, Javitt DC, Heresco-Levy U (2005). Relation of plasma glycine, serine, and homocysteine levels to schizophrenia symptoms and medication type. Am J Psychiatry.

[3] McEvoy JP, Meyer JM, Goff DC (2005). Prevalence of the metabolic syndrome in patients with schizophrenia: baseline results from the Clinical Antipsychotic Trials of Intervention Effectiveness (CATIE) schizophrenia trial and comparison with national estimates from NHANES III. Schizophr Res.

[4] Selhub J (2006). The many facets of hyperhomocysteinemia: studies from the Framingham cohorts. J Nutr.

[5] Saedisomeolia A, Djalali M, Moghadam AM, Ramezankhani O, Najmi L (2011). Folate and vitamin B12 status in schizophrenic patients. J Res Med Sci.

[6] Lee YS, Han DH, Jeon CM, Lyoo IK, Na C, Chae SL, Cho SC (2006). Serum homocysteine, folate level and methylenetetrahydrofolate reductase 677, 1298 gene polymorphism in Korean schizophrenic patients. NeuroReport.

[7] Mattson MP, Shea TB (2003). Folate and homocysteine metabolism in neural plasticity and neurodegenerative disorders. Trends Neurosci.

[8] Dietrich-Muszalska A, Malinowska J, Olas B, Glowacki R, Bald E, Wachowicz B, Rabe-Jablonska J (2012). The oxidative stress may be induced by the elevated homocysteine in schizophrenic patients. Neurochem Res.

[9] Ho PI, Ortiz D, Rogers E, Shea TB (2002). Multiple aspects of homocysteine neurotoxicity: glutamate excitotoxicity, kinase hyperactivation and DNA damage. J Neurosci Res.

[10] Lerner V, Miodownik C, Kaptsan A, Vishne T, Sela BA, Levine J (2005). High serum homocysteine levels in young male schizophrenic and schizoaffective patients with tardive parkinsonism and/or tardive dyskinesia. J Clin Psychiatry.

[11] Levine J, Stahl Z, Sela BA (2006). Homocysteine-reducing strategies improve symptoms in chronic schizophrenic patients with hyperhomocysteinemia. Biol Psychiatry.

[12] Kane JM (2012). Addressing nonresponse in schizophrenia. J Clin Psychiatry.

[13] Newcomer JW (2005). Second-generation (atypical) antipsychotics and metabolic effects: a comprehensive literature review. CNS Drugs.

[14] Auquier P, Lancon C, Rouillon F, Lader M, Holmes C (2006). Mortality in schizophrenia. Pharmacoepidemiol Drug Saf.

[15] Iqbal MM, Rahman A, Husain Z, Mahmud SZ, Ryan WG, Feldman JM (2003). Clozapine: a clinical review of adverse effects and management. Ann Clin Psychiatry.

[16] Okamura N, Hashimoto K, Kanahara N, Shimizu E, Kumakiri C, Komatsu N, Iyo M (2003). Protective effect of the antipsychotic drug zotepine on dizocilpine-induced neuropathological changes in rat retrosplenial cortex. Eur J Pharmacol.

[17] Magliaro BC, Saldanha CJ (2009). Clozapine protects PC-12 cells from death due to oxidative stress induced by hydrogen peroxide via a cell-type specific mechanism involving inhibition of extracellular signal-regulated kinase phosphorylation. Brain Res.

[18] Moller M, Du Preez JL, Emsley R, Harvey BH (2011). Isolation rearing-induced deficits in sensorimotor gating and social interaction in rats are related to cortico-striatal oxidative stress, and reversed by sub-chronic clozapine administration. Eur Neuropsychopharmacol.

[19] Fehsel K, Loeffler S, Krieger K, Henning U, Agelink M, Kolb-Bachofen V, Klimke A (2005). Clozapine induces oxidative stress and proapoptotic gene expression in neutrophils of schizophrenic patients. J Clin Psychopharmacol.

[20] Bosy-Westphal A, Later W, Hitze B (2008). Accuracy of bioelectrical impedance consumer devices for measurement of body composition in comparison to whole body magnetic resonance imaging and dual X-ray absorptiometry. Obes Facts.

[21] Levine J, Stahl Z, Sela BA, Gavendo S, Ruderman V, Belmaker RH (2002). Elevated homocysteine levels in young male patients with schizophrenia. Am J Psychiatry.

[22] Henderson DC, Copeland PM, Nguyen DD (2006). Homocysteine levels and glucose metabolism in non-obese, non-diabetic chronic schizophrenia. Acta Psychiatr Scand.

[23] Sanchez-Margalet V, Valle M, Ruz FJ, Gascon F, Mateo J, Goberna R (2002). Elevated plasma total homocysteine levels in hyperinsulinemic obese subjects. J Nutr Biochem.

[24] Esteghamati A, Mousavizadeh M, Noshad S, Shoar S, Khalilzadeh O, Nakhjavani M (2012) Accuracy of anthropometric parameters in identification of high-risk patients predicted with cardiovascular risk models. Am J Med Sci [Epub ahead of print] 10.1097/MAJ.0b013e31826485de 22986615

[25] Bjorntorp P (1988). The associations between obesity, adipose tissue distribution and disease. Acta Med Scand Suppl.

[26] Lin YH, Pao KY, Yang WS (2008). Waist-to-hip ratio correlates with homocysteine levels in male patients with coronary artery disease. Clin Chem Lab Med.

[27] Battezzati A, Bertoli S, San Romerio A, Testolin G (2007). Body composition: an important determinant of homocysteine and methionine concentrations in healthy individuals. Nutr Metab Cardiovasc Dis.

[28] Abbasi F, Facchini F, Humphreys MH, Reaven GM (1999). Plasma homocysteine concentrations in healthy volunteers are not related to differences in insulin-mediated glucose disposal. Atherosclerosis.

